# Aucubin exerts anti-osteoporotic effects by promoting osteoblast differentiation

**DOI:** 10.18632/aging.102742

**Published:** 2020-02-05

**Authors:** Yutong Li, Yongfeng Zhang, Xinrui Zhang, Wenqian Lu, Xin Liu, Min Hu, Di Wang

**Affiliations:** 1 Department of Orthodontics, School and Hospital of Stomatology, Jilin University, Changchun 130021, China; 2 School of Life Sciences, Jilin University, Changchun 130012, China; 3 Jilin Provincial Key Laboratory of Tooth Development and Bone Remodeling, Changchun 130021, China

**Keywords:** aubucin, osteoporosis, oxidative stress, osteoblast, Nrf2

## Abstract

Osteoporosis is a metabolic disease characterized by reduced osteoblast differentiation and proliferation. Oxidative stress plays a role in the pathogenesis of osteoporosis. Aucubin (AU), an iridoid glycoside, was previously shown to promote osteoblast differentiation. We investigated the effects of AU on MG63 human osteoblast-like cells treated with dexamethasone (Dex) or hydrogen peroxide (H_2_O_2_) to induce oxidative damage. AU protected MG63 cells against apoptosis, and promoted increased expression of cytokines associated with osteoblast differentiation, including collagen I, osteocalcin (OCN), osteopontin (OPN), and osterix. In Dex- and H_2_O_2_-treated MG63 cells, AU also enhanced the expression of anti-oxidative stress-associated factors in the nuclear respiratory factor 2 signaling pathway, including superoxide dismutases 1 and 2, heme oxygenases 1 and 2, and catalase. *In vivo*, using a Dex-induced mouse model of osteoporosis, AU promoted increased cortical bone thickness, increased bone density, and tighter trabecular bone. Additionally, it stimulated an increase in the expression of collagen I, OCN, OPN, osterix, and phosphorylated Akt and Smads in bone tissue. Finally, AU stimulated the expression of cytokines associated with osteoblast differentiation in bone tissue and serum. Our data indicate AU may have therapeutic efficacy in osteoporosis.

## INTRODUCTION

Osteoporosis is a metabolic disease characterized by the destruction of bone tissue and a reduction in total bone mass [1]. It results from an imbalance between osteoblast-mediated bone formation and osteoclast-mediated bone resorption, which is essential for normal bone metabolism [2]. Therapeutics for osteoporosis have predominantly targeted osteoclasts to prevent bone resorption. However, they can result in serious adverse effects [3]. For example, bisphosphonates can cause jaw necrosis, esophageal cancer, and renal failure. Parathyroid hormone (PTH) is the only Food and Drug Administration-approved agent that stimulates bone formation, but it has been linked to osteosarcoma and can only be used for 2 years [4].

The production of reactive oxygen species (ROS) through mitochondrial respiration increases with age. The accumulation of ROS causes intracellular oxidative stress [5], which plays a role in the pathogenesis of osteoporosis. Oxidative stress can induce cell apoptosis and disrupt the balance between osteoblast and osteoclast activity. This can lead to reduced proliferation and differentiation of osteoblasts from bone marrow mesenchymal stem cells, which reduces bone formation and bone mass [6–8]. Nuclear respiratory factor 2 (Nrf2) is required for the induction of superoxide dismutase (SOD) and activation of the antioxidant response to internal and external chemical stimuli [9]. Interestingly, a statin (Simvastatin) demonstrated anti-osteoporotic effects by increasing heme oxygenase (HO-1) and SOD levels thereby reducing oxidative stress [10].

Several natural compounds have been identified that exhibit anti-osteoporotic effects without causing adverse events and toxicities [11]. Three categories of agents have been identified: (1) phytoestrogens with estrogenic effects, (2) antioxidants and anti-inflammatory agents, and (3) compounds with pleiotropic effects [12]. Aucubin (AU) is an iridoid glycoside compound primarily derived from *Eucommia ulmoides* that has anti-osteoporotic effects [13]. It was previously found to promote angiogenesis, and displayed hepatoprotective, anti-inflammatory, and anti-oxidative effects [14]. It was also shown to promote embryonic hippocampal neural stem cell differentiation in rats [15]. We previously demonstrated that AU could promote osteoblast differentiation by regulating bone morphogenetic protein-2 (BMP2) [16]. Therefore, we hypothesized that AU could have therapeutic efficacy for osteoporosis.

In this study, we investigated the effects of AU on human osteoblast-like cells treated with dexamethasone (Dex) or hydrogen peroxide (H_2_O_2_) to induce oxidative damage, and in a Dex-induced mouse model of osteoporosis.

## RESULTS

### AU protected MG63 cells against Dex-induced damage via modulation of Nrf2 signaling

**Figure 1:**
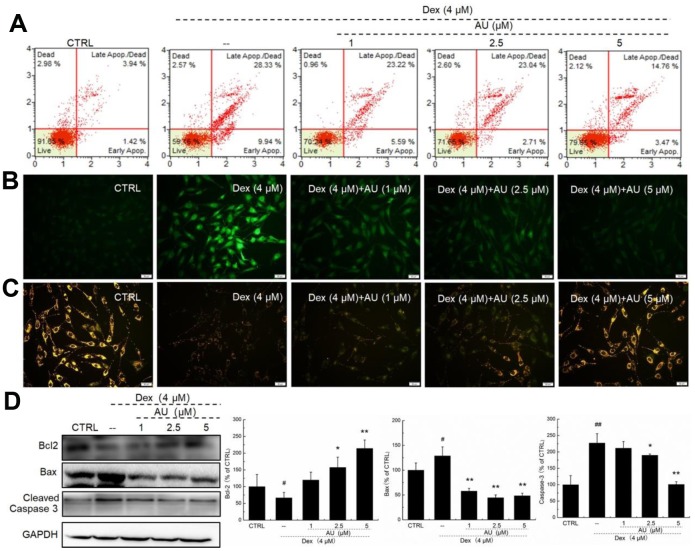
**AU protected MG63 cells against Dex damage.** (**A**) AU reduced the apoptosis rate of MG63 cells caused by Dex after 24 h incubation. (**B**) AU suppressed the over-accumulation of ROS in MG63 cells caused by Dex after 24 h incubation. (**C**) AU inhibited the dissipation of MMP in MG63 cells caused by Dex. (**D**) AU enhanced the expression levels of Bcl-2, and reduced the expression levels of Bax and cleaved caspase-3 in MG63 cells exposed to Dex. The quantification data of the expression levels of Bcl2, casepase3 and Bax were normalized by corresponding GAPDH. Data are expressed as mean ± S.D. (n=6) and analyzed using a one-way ANOVA. # *P*<0.05 and ## *P*<0.01 *vs.* control cells, *P<0.05 and **P<0.01 *vs.* Dex-exposed cells.

**Figure 2:**
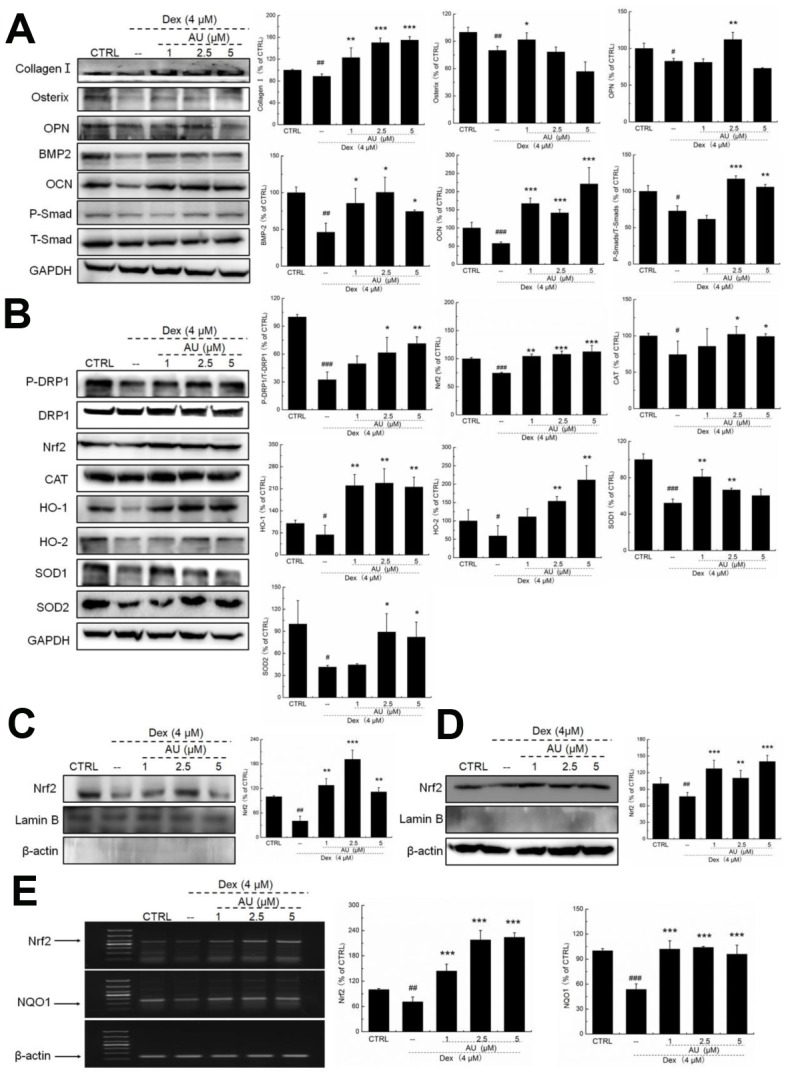
**AU protected the Dex-caused MG63 cells apoptosis via regulation the Nrf2/HO-1 signaling.** (**A**) AU up-regulated the expression levels of osteoblast differentiation related proteins including Osterix, OPN, BMP2, OCN and P-Smad in MG63 cells exposed to Dex. (**B**) AU increased the expression levels of proteins within the Nrf2/HO-1 signaling including P-DPR1, Nrf2, CAT, HO-1, HO-2, SOD-1 and SOD-2 in MG63 cells exposed to Dex. AU enhanced the expression levels of Nrf2 in both (**C**) nucleus and (**D**) cytoplasm of MG63 cells exposed to Dex. The quantification data of proteins were normalized by corresponding GAPDH and total proteins, respectively (n=4). (E) AU increased the mRNA levels of Nrf2 and NQO-1 in MG63 cells exposed to Dex. Marker size from top to bottom: 1000 bp, 700 bp, 500 bp, 400 bp, 300 bp, 200 bp and 100 bp. The data on quantified mRNA expression were normalized to the levels of β-actin (n=4). Data are expressed as mean ± S.D. and analyzed using a one-way ANOVA. # *P*<0.05, ## *P*<0.01 and ### *P*<0.001 *vs.* control cells, *P<0.05, **P<0.01 and ***P<0.001 *vs.* Dex-exposed cells.

AU reduced the apoptotic rate of MG63 cells exposed to 4 μM of Dex for 24 h in a dose-dependent manner (Figure 1A). Mitochondrial function is one of the factors contributing to apoptosis and it plays a role in the feedback loop that responds to ROS accumulation [17]. The over-accumulation of intracellular ROS (Figure 2B) and the enhanced dissipation of MMP (Figure 2C) in MG63 cells caused by Dex were all strongly relieved by AU at doses of 1, 2.5 and 5 μM, as shown by the reduced green fluorescence intensity, and enhanced ratio of red/green fluorescence intensity, respectively. Bcl-2 family members contribute to cell apoptosis related to mitochondrial function [15]. Compared to MG63 cells exposed to Dex alone, AU significantly enhanced the expression levels of Bcl-2 and reduced the expression levels of Bax and cleaved caspase-3 (*P* <0.05) (Figure 1D).

According to our study and previous findings, proteins including collagen I, osterix, OPN, BMP-2, OCN, and Smads are biomarkers of osteoblast differentiation [16]. 24-h 4 μM DEX exposure strongly reduced the expression levels of all proteins related to osteoblast differentiation, including collagen I, osterix, OPN, BMP-2, OCN, and P-Smads (*P* <0.05) (Figure 2A), which were all up-regulated after AU incubation (*P* <0.05) (Figure 2A).

In Dex-alone exposed MG63 cells, the levels of anti-oxidative proteins related to Nrf2 signaling were strongly reduced (*P* <0.05) (Figure 2B). Compared with Dex-damaged cells, AU treatment resulted in 89.8%, 40.2%, 22.5% 237.2%, 159.1%, 27.6%, and 98.5% increases in the expression levels of PDRP1, Nrf2, CAT, HO-1, HO-2, SOD-1, and SOD-2 in MG63 cells, respectively (*P* <0.05) (Figure 2B). Furthermore, in Dex-damaged MG63 cells, AU increased the expression levels of Nrf2 in both nucleus (*P* <0.001) (Figure 2C) and cytoplasm (*P* <0.001) (Figure 2D). Compared with Dex-damaged cells, AU enhanced the mRNA levels of Nrf2 (*P* <0.001) and NAD(P)H dehydrogenase [quinone] 1 (NQO1) (*P* <0.001) in MG63 cells (Figure 2E).

### AU protected MG63 cells against H_2_O_2_-induced damage related to Nrf2 signaling

**Figure 3:**
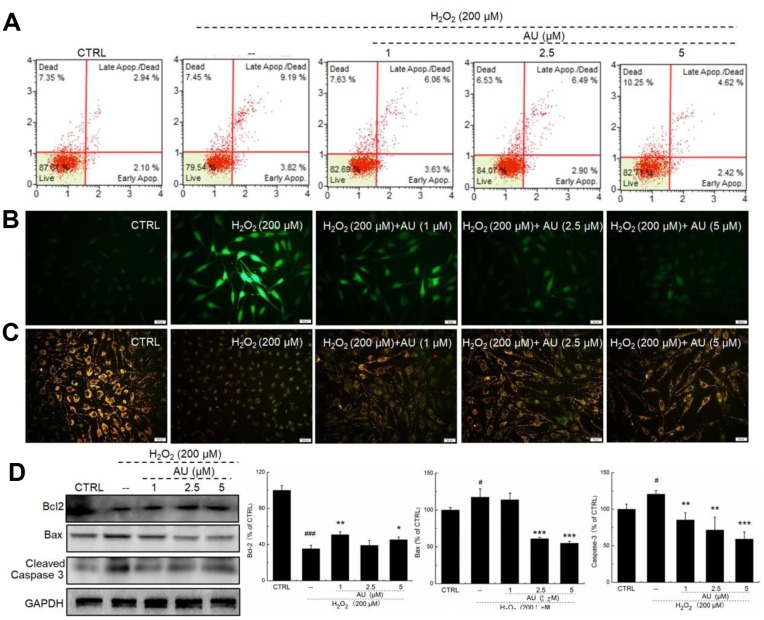
**AU restored the damage of H_2_O_2_ on MG63 cells.** (**A**) AU reduced the apoptosis rate of MG63 cells caused by H_2_O_2_. (**B**) AU suppressed the over-accumulation of ROS in MG63 cells caused by H_2_O_2_. (**C**) AU inhibited the dissipation of MMP in MG63 cells caused by H_2_O_2_. (**D**) AU enhanced the expression levels of Bcl-2, and reduced the expression levels of Bax and cleaved caspase-3 in MG63 cells exposed to H_2_O_2_. The quantification data of the expression levels of Bcl2, casepase3 and Bax were normalized by corresponding GAPDH. Data are expressed as mean ± S.D. (n=6) and analyzed using a one-way ANOVA. # *P*<0.05 and ### *P*<0.001 *vs.* control cells, *P<0.05, *P<0.01 and ***P<0.001 H_2_O_2_-exposed cells.

In H_2_O_2_-induced apoptotic MG63 cells, AU was protective against H_2_O_2_ damage via reducing the apoptosis rate (Figure 3A), suppressing the accumulation of ROS (Figure 3B), and inhibiting the dissipation of MMP (Figure 3C). Compared with H_2_O_2_-damaged MG63 cells, AU incubation resulted in a 13.6% increase in the expression of Bcl-2 (*P* <0.01; Figure 3D), and 25.7% and 29.2% reductions in the expression of Bax (*P* <0.001) (Figure 3D) and cleaved caspase-3 (*P* <0.01) (Figure 3D).

**Figure 4:**
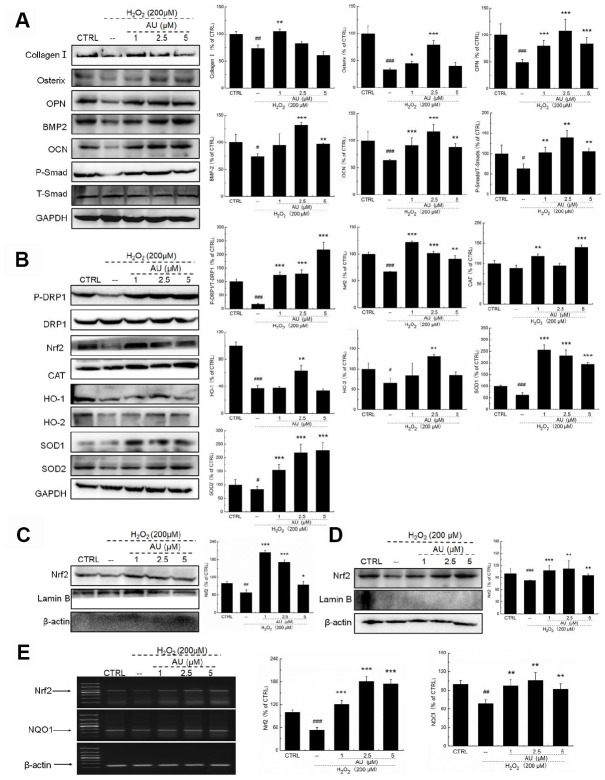
**AU protected the H_2_O_2_-caused MG63 cells apoptosis via regulation the Nrf2/HO-1 signaling.** (**A**) AU up-regulated the expression levels of osteoblast differentiation related proteins including Osterix, OPN, BMP2, OCN and P-Smad in MG63 cells exposed to H_2_O_2_. (**B**) AU increased the expression levels of proteins within the Nrf2/HO-1 signaling including P-DPR1, Nrf2, CAT, HO-1, HO-2, SOD-1 and SOD-2 in MG63 cells exposed to H_2_O_2_. AU enhanced the expression levels of Nrf2 in both (**C**) nucleus and (**D**) cytoplasm of MG63 cells exposed to H_2_O_2_. The quantification data of proteins were normalized by corresponding GAPDH and total proteins, respectively (n=4). (**E**) AU increased the mRNA levels of Nrf2 and NQO-1 in MG63 cells exposed to H_2_O_2_. Marker size from top to bottom: 1000 bp, 700 bp, 500 bp, 400 bp, 300 bp, 200 bp and 100 bp. The data on quantified mRNA expression were normalized to the levels of β-actin (n=4). Data are expressed as mean ± S.D. and analyzed using a one-way ANOVA. # *P*<0.05, ## *P*<0.01 and ### *P*<0.001 *vs.* control cells, *P<0.05, **P<0.01 and ***P<0.001 *vs.* Dex-exposed cells.

Compared with H_2_O_2_-damaged MG63 cells, AU up-regulated the expression of biomarkers of osteoblast differentiation, including collagen I, osterix, OPN, BMP-2, OCN, and P-Smads by 42.3%, 34.7%, 62.3%, 30.6%, 38.5%, and 62.3%, respectively (*P* <0.05) (Figure 4A). AU resulted in 654.5%, 35.9%, 33.4%, 69.5%, 99.1%, 213.7%, and 85.0% increases in the expression of PDRP1, Nrf2, CAT, HO-1, HO-2, SOD-1, and SOD-2 in H_2_O_2_-damaged MG63 cells (*P* <0.05) (Figure 4B). Furthermore, in H_2_O_2_-damaged MG63 cells, AU increased the expression levels of Nrf2 in both nucleus (*P* <0.01) (Figure 4C) and cytoplasm (*P* <0.01) (Figure 4D). Compared with H_2_O_2_-damaged cells, AU increased the mRNA levels of Nrf2 (*P* <0.001) and NQO1 (*P* <0.01) in MG63 cells (Figure 4E).

### AU protected the Dex-damaged mice against osteoporosis

**Figure 5:**
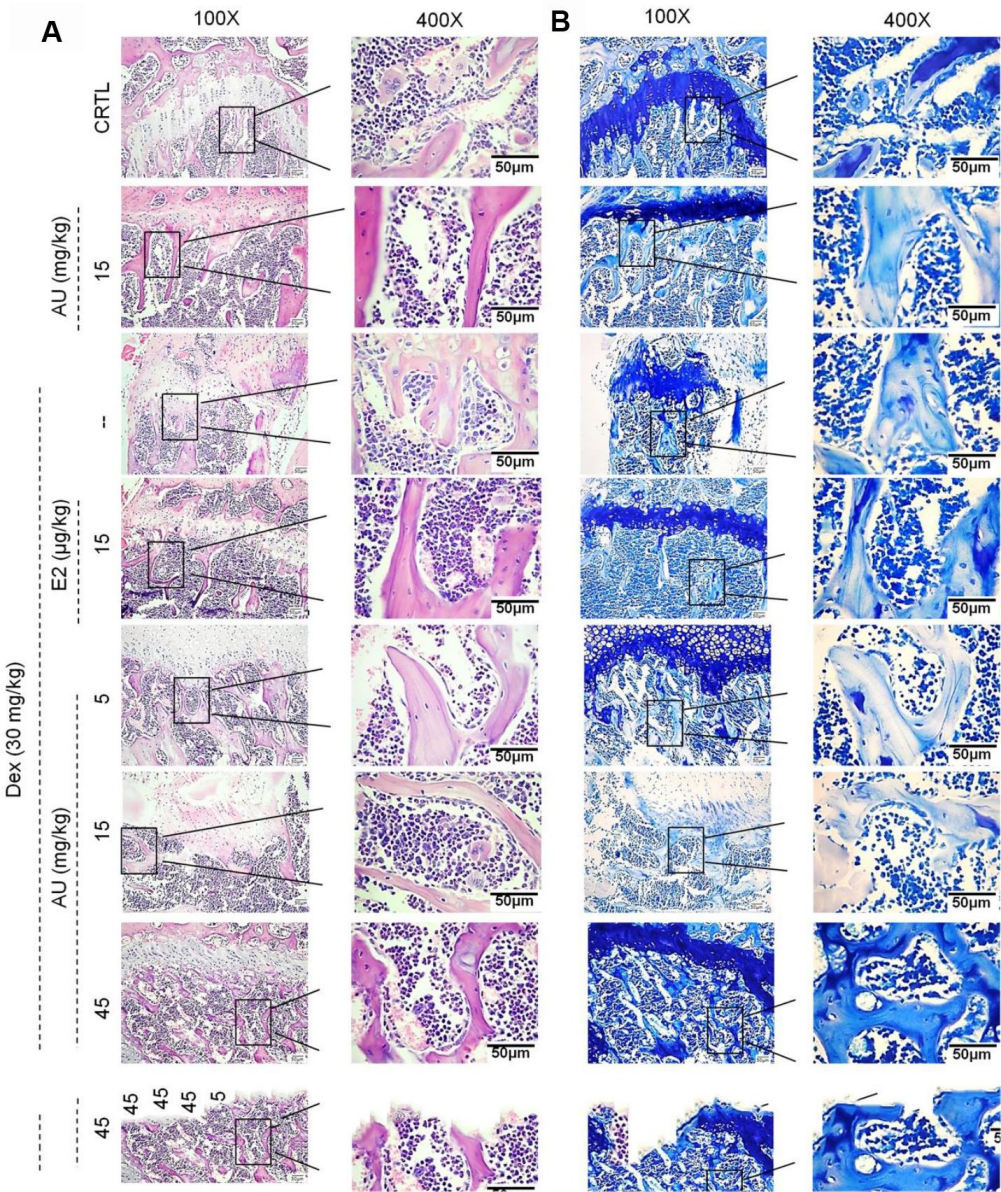
The effects of AU on the femoral histological changes of osteoporotic mice were detected by (**A**) H&E staining and (**B**) Giemsa staining (n=6).

Compared with Dex-damaged mice with osteoporosis, 8-week AU administration made the cortical bone more continuous and reduced the number of osteoclasts, as detected by HȦE staining (Figure 5A). Giemsa staining revealed that AU treatment enhanced the number of trabecular osteoblasts in the mice (Figure 5B).

**Figure 6:**
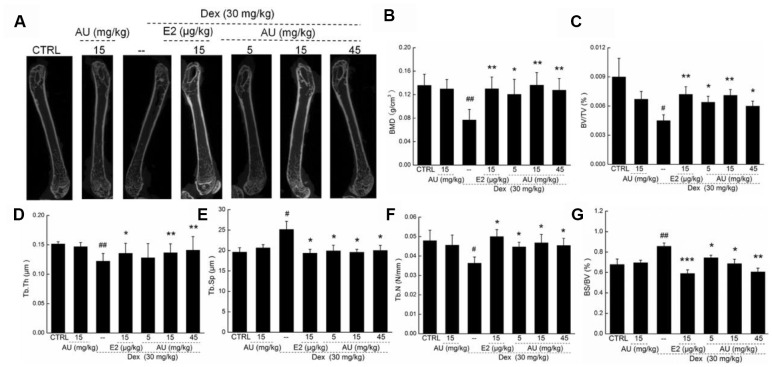
**The effects of AU on the femoral bone morphological changes and the levels of osteoporotic indexes of osteoporotic mice.** (**A**) The photographs of femur in osteoporotic mice detecting via micro-CT. The levels of (**B**) BMD, (**C**) BV/TV, (**D**) Tb.Th, (**F**) Tb.N and (**G**) BS/BV among all groups were analyzed. Data are expressed as mean ± S.D. (n=6) and analyzed using a one-way ANOVA. # *P*<0.05 and ## *P*<0.01 *vs.* CTRL mice, *P<0.05 and **P<0.01 Dex-treated mice.

The structural parameters of femur trabecular and cortical regions, and of the tibia cortical region, were detected via micro-CT (Figures 6 and Supplementary Figure 1). Compared with the control mice, thinner cortical bones and sparser trabecular bone were observed in the model mice with osteoporosis (Figure 6A). Comparatively, E2 and AU enhanced the thickness of the bone cortex and the density of the trabecular bone, as shown by the increased brightness (Figure 6A). Using standard 3D microstructural analysis, the bone mineral density (BMD), bone volume fraction (BV/TV), trabecular thickness (Tb.Th), trabecular spacing (Tb.Sp) and trabecular number (Tb.N) were calculated for each group. Compared with the control mice, reduced levels of BMD (*P* <0.01) (Figure 6B), BV/TV (*P* <0.05) (Figure 6C), Tb.Th (*P* <0.05; Figure 6D) and Tb.N (*P* <0.05) (Figure 6F), and the increased levels of Tb.Sp (*P* <0.05) (Figure 6F) and BS/BV (*P* <0.05) (Figure 6G) were noted in Dex-damaged mice with osteoporosis. Similar to the effects of E2, 8-week AU administration significantly enhanced the levels of BMD (*P* <0.01) (Figure 6B), BV/TV (*P* <0.05) (Figure 6C), Tb.Th (*P* <0.01) (Figure 6D), and Tb.N (*P* <0.05) (Figure 6F), and reduced the levels of Tb.Sp (*P* <0.05) (Figure 6F) and BS/BV (*P* <0.05; Figure 6G) in the osteoporotic mice.

Administration of AU for 8 weeks at 15 mg/kg had no significant effects on the bone structures of healthy mice compared with the control mice (Figures. 5 and 6).

### AU promoted osteoblast differentiation in osteoporotic mice

Dex injection caused a significant reduction in the levels of factors related to osteoblast differentiation, including ALP, collagen I, OCN, OPN, BMP2 and BMPR2, and in the levels of Ca, Pi and E2 in the peripheral blood of Dex-injected mice with osteoporosis (*P* <0.05) (Table 1), which were strongly enhanced after 8 weeks’ AU and E2 administration (*P* <0.05) (Table 1). Administration of AU for 8 weeks at 15 mg/kg had no significant effects on the serum levels of these factors (Table 1).

**Table 1:** The effects of AU on osteoblast differentiation related factors in peripheral blood of Dex-injected mice with osteoporosis.

**Groups**	**CTRL**	**AU (15mg/kg)**	**--**	**E2 (15 μg/kg)**	**Dex(30mg/kg)**		
					**AU (mg/kg)**		
					**5**	**15**	**45**
Ca (mmol/L)	1.5±0.1	1.4±0.1	1.3±0.1^#^	1.6±0.1**	1.5±0.1*	1.5±0.1*	1.5±0.1*
Pi (mmol/L)	2.3±0.2	2.4±0.2	2.1±0.1^#^	2.4±0.2**	2.4±0.2*	2.4±0.3*	2.2±0.2
E2 (pmol/L)	45.1±2.5	44.4±1.3	40.6±1.2^#^	44.5±2.5*	44.2±3.3	44.0±6.0	51.3±0.1**
ALP (IU/L)	8.1±1.8	7.7±1.5	5.1±1.0^##^	7.5±1.3**	6.6±1.0*	7.7±0.8**	6.4±0.3**
Collagen I (ng/mL)	5.2±0.6	5.1±0.6	4.1±0.4^##^	4.8±0.7*	5.0±0.7*	5.7±0.8***	5.6±0.7***
OCN (ng/mL)	2.4±0.2	2.6±0.3	2.0±0.2^##^	2.3±0.2*	2.3±0.1**	2.6±0.3**	2.3±0.2*
OPN (ng/mL)	43.3±3.3	41.2±3.5	36.4±2.3^#^	42.7±4.0*	41.5±1.5*	42.4±5.7*	44.3±5.4*
BMP-2 (ng/mL)	2.3±0.2	2.2±0.2	1.8±0.2^##^	2.0±0.2*	1.9±0.3	2.0±0.5	2.4±0.3**
BMPR-2 (ng/mL)	1.2±0.1	1.1±0.1	0.9±0.1^##^	1.1±0.1*	1.2±0.1**	1.2±0.1**	1.3±0.1**

The data were analyzed using a one-way ANOVA and expressed as means ± S.E.M. (n = 10). # *P* < 0.05 and ## *P* < 0.01 versus control mice; * *P* < 0.05, ** *P* < 0.01 and ***P < 0.001 versus osteoporosis injured mice.

**Figure 7:**
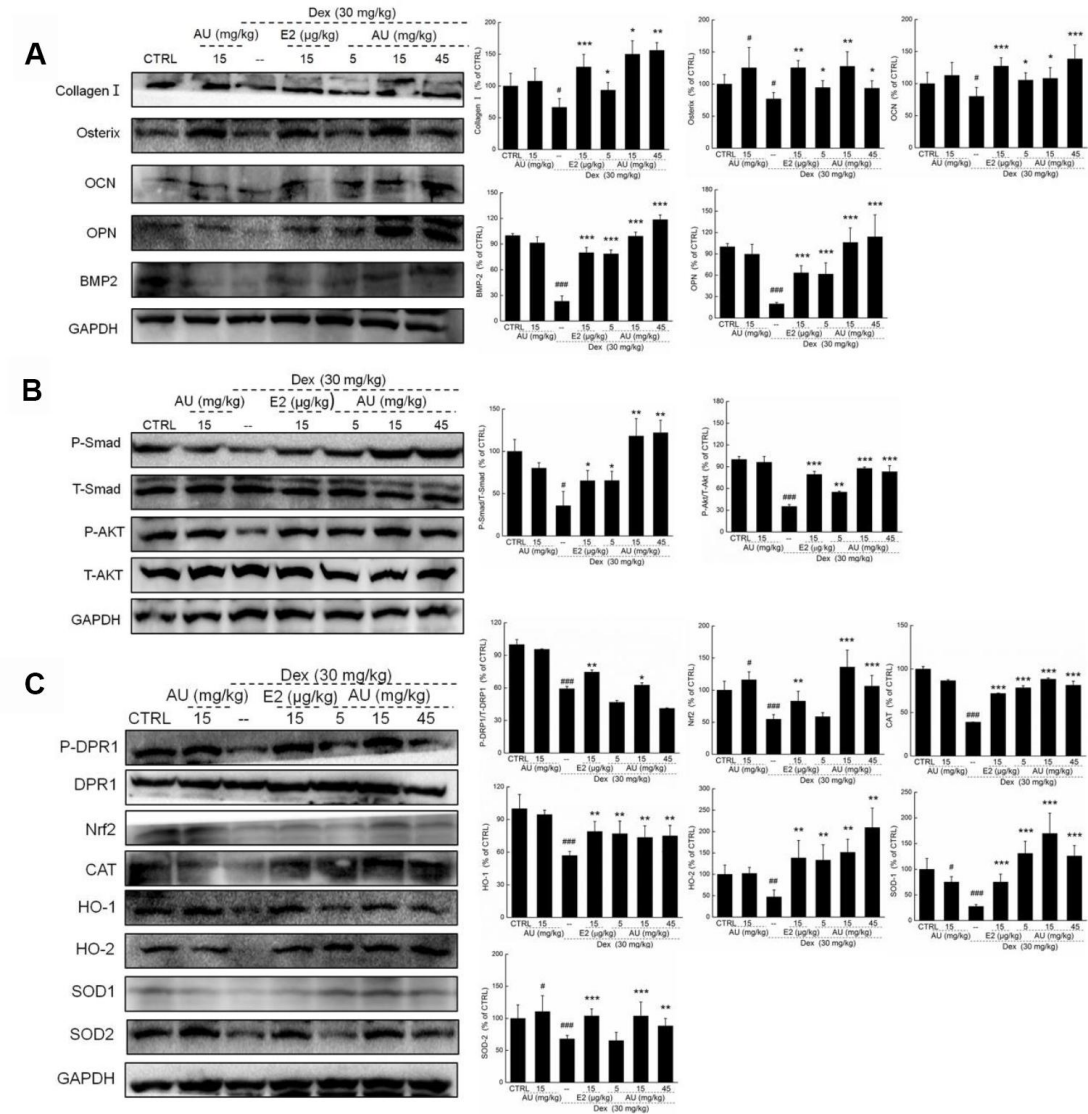
**AU enhanced the osteoblast differentiation of femoral bone in Dex-ineduced osteoporotic mice.** (**A**) AU enhanced the expression levels of osteoblast differentiation related proteins including collagen I, Osterix, OCN, OPN, BMP2, and P4HB in femoral bone tissues of Dex-ineduced osteoporotic mice. (**B**) AU enhanced the expression levels of P-Smad and P-Akt in femoral bone tissues of Dex-ineduced osteoporotic mice. (**C**) The effects of AU on the expression levels of Nrf2/HO-1 signaling related proteins in femoral bone tissues of Dex-ineduced osteoporotic mice, including P-DRP1, Nrf2, CAT, HO-1, HO-2, SOD-1, SOD-2, P-Smad and P-Akt. The quantification data of proteins were normalized by corresponding GAPDH and total proteins, respectively, expressed as mean ± S.D. (n=6) and analyzed using a one-way ANOVA. # *P*<0.05, ## *P*<0.01 and ### *P*<0.001 *vs.* CTRL mice, *P<0.05, **P<0.01 and ***P<0.001 Dex-treated mice.

Different from E2, AU showed no significant effects on the levels of TRACP-5b (Supplementary Figure 2A). AU at 15 mg/kg and E2 at 15 μg/kg strongly enhanced the levels of TNF-α compared with model mice (*P*<0.01) (Supplementary Figure 2B) Similarly, remarkably low expression levels of collagen I, osterix, OCN, OPN, BMP2, P-Smads and P-Akt were noted in the lysed tibias and fibulas of Dex-induced osteoporotic mice (*P*<0.05) (Figure 7A and 7B). Both E2 and AU treatment increased all of the detected proteins (*P* <0.05) (Figure 7A and 7B). AU administration resulted in 40.5%, 21.6%, 31.5%, 112.9%, 238.7%, 83.1%, and 56.2% increases in the expression levels of collagen I, osterix, OCN, OPN, BMP2, P-Smads and P-Akt, respectively, at 8 weeks (*P* <0.05) (Figure 7A and 7B). Among all of the detected proteins, administration of AU alone strongly enhanced the expression of osterix (*P* <0.05), but had no significant effects on other factors in the bone tissues of healthy mice compared with the control group (Figure 7A).

### Nrf2 signaling is involved in AU-mediated anti-osteoporotic activity

**Figure 8:**
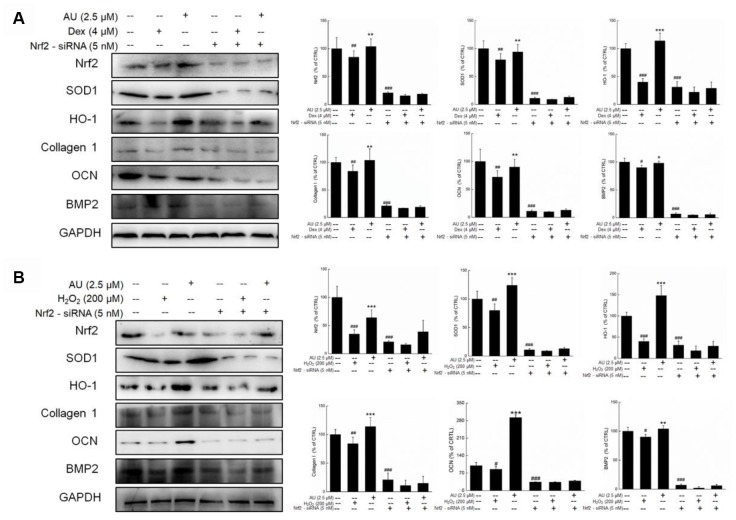
**The Nrf2-siRNA transfection strongly abolished the effects of AU on the protein expressions in Dex and H_2_O_2_ damaged MG63 cells.** The up-regulation of AU on the expressions of Nrf2, SOD1, HO-1, OCN and BMP were strongly abolished in Nrf2-siRNA transfected MG63 cells after (**A**) Dex and (**B**) H_2_O_2_ exposure. The quantification data of proteins were normalized by corresponding GAPDH, respectively, expressed as mean ± S.D. (n=4) and analyzed using a one-way ANOVA. # *P*<0.05, ## *P*<0.01 and ### *P*<0.001 *vs.* control cells, *P<0.05, **P<0.01 and ***P<0.001 *vs.* Dex or H_2_O_2_-exposed cells.

It is well known that oxidative stress inhibits bone cell differentiation and impairs bone integrity [18]. In Dex-induced osteoporotic mice, high levels of ROS and low levels of SOD and CAT were noted in peripheral blood (*P* <0.05) (Table 2). Compared with the osteoporotic mice, AU resulted in an 18.0% reduction in ROS, and 15.6% and 35.1% increases in SOD and CAT levels in peripheral blood (*P* <0.05) (Table 2). In the lysed tibias and fibulas of the Dex-induced osteoporotic mice, the expression levels of Nrf2 signaling proteins were all strongly reduced (*P* <0.05) (Figure 8). Comparatively, E2 and AU relieved these reductions (*P* <0.05) (Figure 8). AU, especially at doses of 15 mg/kg, caused 16.4%, 94.5%, 101.2%, 12.5%, 182.3%, 173.6%, and 30.2% increases in the expression levels of P-DPR1, Nrf2, CAT, HO-1, HO-2, SOD-1, and SOD-2 in bone tissues compared with those of the control mice (*P* <0.05) (Figure 7C).

**Table 2:** The effects of AU on the oxidative stress related factors in peripheral blood of Dex-injected mice with osteoporosis.

	**CTRL**	**AU (15mg/kg)**	**Dex (30mg/kg)**				
			**--**	**E2 (15 μg/kg)**	**AU (mg/kg)**		
					**5**	**15**	**45**
ROS (U/mol)	50.7±4.4	49.2±4.1	58.3±1.3^#^	45.5±4.1**	47.8±2.0**	46.0±1.8**	47.1±0.5**
CAT (U/mL)	171.6±30.9	162.3±36.0	137.3±21.1^#^	201.5±20.5**	199.4±37.2**	189.0±40.0*	185.5±40.5*
SOD (U/mL)	277.5±16	266.6±21.9	240.2±20.4^#^	285.7±16.1*	277.7±10.8*	310.2±22.8**	278.8±20.3*

The data were analyzed using a one-way ANOVA and expressed as means ± S.E.M. (n = 10). # P < 0.05 and ## P < 0.01 versus control mice; * P < 0.05 and ** P < 0.01 versus osteoporotic mice.

Further experiments were performed in Nrf2-siRNA transfected MG63 cells. The enhancing effects of AU on the expressions of Nrf2, SOD1, HO-1, Collagen 1, OCN, and BMP2 were all suppressed by Nrf2-siRNA transfection in both Dex and H_2_O_2_ damaged transfected MG63 cells (Figure 8A and 8B). In contrast, the negative siRNA transfection failed to influence the modulatory effects of AU on the expressions of Nrf2, SOD1, HO-1, Collagen 1, OCN, and BMP2 in both Dex and H_2_O_2_ damaged MG63 cells (Supplementary Figure 3A and 3B).

## DISCUSSION

Bone structure and quality are the main factors that affect strength and performance [19]. Thin and discontinuous bony cortices are typically observed in patients with osteoporosis [20]. The degree of trabecular mineralization (equivalent to the density of the mineral deposited in collagen) is frequently evaluated in osteoporosis patients as an index of bone turnover and the mechanical properties of the bone [21]. Our findings indicate that AU can enhance bone toughness and density, thicken the bone cortex, increase the mineralization of the bone trabeculae, and decrease the size of the mesh in a Dex-induced mouse model of osteoporosis.

Oxidative stress can promote the development of osteoporosis [22, 23]. The intracellular accumulation of ROS causes mitochondrial dysfunction and can induce apoptosis [24, 25]. ROS accumulation has been observed in bone tissue from patients with degenerative diseases such as osteoporosis [26]. Oxidative stress caused by estrogen deficiency or inflammatory bone disorders contributes to osteoporosis and bone resorption [22, 27]. Here, we found that AU reduced the expression of Bax and cleaved caspase-3, increased the expression of Bcl-2, and reduced the rate of apoptosis in Dex- and H_2_O_2_-treated MG63 cells. AU also inhibited ROS production and prevented dissipation of the mitochondrial membrane potential (MMP). Bcl-2 and Bax, which are located in the mitochondrial membrane, inhibit the production of oxygen free radicals and act as antioxidants to protect against mitochondrial apoptosis [28, 29]. Under conditions of oxidative stress, the rate of apoptosis among mature bone cells increases and can contribute to osteoporosis [30]. Antioxidants protect bone cells against oxidative stress by inducing osteoblastogenesis and inhibiting osteoclast activation [31].

Oxidative stress inhibits osteoblast differentiation and promotes osteoclast differentiation [32]. We observed a reduction in the expression of proteins associated with osteoblast differentiation in Dex- and H_2_O_2_-treated MG63 cells and in bone tissue from mice with osteoporosis. Osteoblasts play an important role in the formation of the bone matrix and regulation of the bone resorption activities of osteoclasts. Osteoblasts synthesize and secrete cytokines such as osteopontin (OPN), alkaline phosphatase (ALP), and osteocalcin (OCN) to regulate osteoclast activity [33]. ALP is secreted by osteoblasts at an early stage during differentiation and regulates the synthesis of collagen I and other non-collagenous bone matrix proteins [34]. ALP is also responsible for the reorganization of mineralization components in the extracellular matrix [35].

Osterix, another marker of osteoblast differentiation, activates OCN in mature osteoblasts and regulates the final stages of bone formation [36, 37]. BMP2 is important for bone formation and reconstruction. It induces the differentiation of mesenchymal cells into bone-forming cells, stimulates the expression of OCN, collagen I, and ALP, and activates Smad and non-Smad signaling by combining with transmembrane Ser/Thr kinase receptors [38]. BMP2 activation results in an increase in phosphorylated Smad1, Smad5, and Smad8 levels. BMP2 also activates ALP and OCN [39]. Activated Smads transmit signals from BMPs from the cytoplasm to the nucleus where they regulate the transcription of target genes [40, 41]. Our results indicate that AU increases the expression of Smads in MG63 cells and in bone tissue from mice with osteoporosis. This suggest that they may have anti-osteoporotic effects in addition to promoting osteoblast differentiation.

Under conditions of oxidative stress, heterodimerization of Keap1 sequesters most Nrf2 in the cytoskeleton. Keap1 has a cysteine-rich surface that is oxidized in response to oxidative and nitrosative stress [42]. Oxidative stress occurs causes the release of Nrf2 from Keap1 and translocation of Nrf2 to the nucleus, where it regulates the downstream antioxidant enzyme gene NQO1, and enhances the tolerance of cells to oxidative stress via influencing the expression of SOD, CAT and HO-1 [43–46]. High levels of Dex cause an increase in ROS [47]. This results in persistent oxidative stress and cellular damage, which contributes to the pathogenesis of osteoporosis [48]. High levels of Nrf2 suppress the production of ROS [42]. Nrf2 deficiency stimulates osteoclast differentiation and activity as a result of increased oxidant production and activation of nuclear factor of activated T-cells (NFAT), which leads to bone resorption [49]. We found that AU suppresses ROS production and decreases the levels of phosphorylated dynamin-related protein 1 (P-DPR1), CAT, HO-1, HO-2, SOD-1, and SOD-2 by increasing the expression of Nrf2 in bone tissue Dex-induced osteoporotic mice. The increase in phosphorylated AKT results in an increase in Nrf2 and phosphorylated Smad [50]. Nrf2 knock-down in MG63 cells abolished the effects of AU, suggesting that AU exerts anti-osteoporotic effects by regulating Nrf2 signaling in response to oxidative stress. Thus, AU may have therapeutic efficacy for osteoporosis and other disorders involving bone remodeling.

## CONCLUSIONS

Our study first confirmed that the anti-osteoporotic property of AU in Dex/H_2_O_2_ exposed MG63 cells and Dex-injected C57BL/6 mice with osteoporosis is due to regulation of Nrf2-medaited oxidative stress. The findings provide experimental evidence that AU may be used to treat diseases associated with bone formation.

## MATERIALS AND METHODS

### Cell culture

MG63 human osteoblast-like cells (CRL-1427, passage < 10) were obtained from the American Type Culture Collection. The cells were cultured in Dulbecco’s Modified Eagle’s Medium (DMEM) supplemented with 10% fetal bovine serum, 100 U / mL penicillin, and 100 μg / mL streptomycin at 37°C in a humidified incubator with 5% CO_2_. All cell culture reagents were obtained from Gibco BRL (USA).

### Measurement of cell apoptosis, MMP, and intracellular ROS

MG63 cells were seeded into 6-well plates at a density of 2 × 10^5^ cells / well. The cells were then treated with 1, 2.5, or 5 μM AU for 2 h followed by 4 μM Dex or 200 μM of H_2_O_2_ for 24 h. Following the incubation, the cells were harvested, resuspended in solution at a concentration of 1×10^6^ cells / mL, and stained with propidium iodide and/or Annexin V for 20 min at room temperature. The rate of apoptosis was measured using a Muse Cell Analyzer (Millipore, USA).

The MMP was evaluated by staining the cells with 2 μmoL / L 5,5’,6,6’-Tetrachloro-1,1’,3,3’-tetraethylbenzimidazolylcarbocyanine iodide (JC-1; Sigma-Aldrich, USA) at 37°C for 15 min in the dark. The cells were then washed three times with phosphate buffered saline (PBS) and red and green fluorescence recorded using a fluorescence microscope (20×; CCD camera, TE2000, Nikon, Japan).

Intracellular ROS was quantified by staining the cells with 10 μM 2’-7’-dichlorodihydrofluorescein diacetate (DCFH-DA, Sigma-Aldrich) for 15 min at 37°C in the dark. The cells were then washed three times with PBS and green fluorescence, which reflects the intracellular ROS level, recorded using a Nikon Eclipse TE 2000-S fluorescence microscope (Nikon, Japan).

### Transfection of siRNA

MG63 cells were seeded into 6-well plates (2 × 10^5^ cells/well). The cells were then transiently transfected with 20 nM Nrf2-siRNA (5’-GGATGAAGAGACCGGAGAA-3’) (R10043.8, RiboBio, China) for 30 min using the riboFECT^TM^CP Reagent (RiboBio) according to the manufacturer’s protocol. Following transfection, the cells were treated with 2.5 μM AU for 2 h and then incubated with either 4 μM Dex or 200 μM H_2_O_2_ for 24 h. The cells were harvested and the expression of Nrf2, SOD1, HO-1, Collagen I, OCN, BMP2, and glyceraldehyde 3-phosphate dehydrogenase (GAPDH) analyzed by western blotting.

### Animal experiments

All animal protocols were approved by the Animal Ethics Committee of Jilin University (20160809). C57BL/6 mice (6–8 weeks old; 18–22 g) were obtained from Yis Laboratory Animal Technology Co., Ltd. (China). Mice were maintained at 23 ± 1°C with a 12-h photoperiod. Food and tap water were provided *ad libitum*. A total of 140 male mice were randomly divided into seven groups (n = 20). Control mice were injected intraperitoneally and intragastrically with 10 mL / kg of 0.9% normal saline every other day for 8 weeks. AU-treated mice were intraperitoneally injected with 10 mL/kg of 0.9% normal saline and intragastrically injected with 15 mg / kg AU every other day for 8 weeks. Osteoporotic mice were generated by intraperitoneal injection of 30 mg / kg of Dex sodium phosphate every other day for 8 weeks. The osteoporotic mice were intragastrically injected with either 10 mL / kg of 0.9% normal saline (n = 20) (model mice), or 5 mg / kg (n = 20), 15 mg / kg (n = 20), or 45 mg / kg (n = 20) AU, and intraperitoneally injected with 15 μg / kg estradiol (E2) (n = 20) (positive control mice) every other day for 8 weeks. The bodyweights of the mice were recorded on days 1, 4, 11, 18, 25, 32, 39, 48, and 55. Mice were euthanized after the last treatment, and tibia and femur tissue collected and weighed immediately.

### Cytokine detection

Peripheral blood was collected from the caudal veins of the mice. The levels of plasma cytokines including ALP (YY02947B), BMP2 (YY02939B), BMP receptor type II (BMPR-2; YY02936B), OCN (YY03287B), OPN (YY03172B), collagen I (YY02771B), ROS (CK-E91516M), tumor necrosis factor α (TNF-α; YY02868B), E2 (YY03302B), platelet activating factor (PAF; S0000-96T), tartaric acid phosphatase 5b (TRACP-5b; YY03144B), SOD (YY03125B), malonaldehyde (MDA; YY03124B), catalase (CAT) (CK-E92636M), Ca^2+^ (C004-2), and Pi (C006) detected using enzyme-linked immunosorbent assays according to the manufacturer’s protocols (Shanghai Yuanye Biological Technology Co., Ltd, China).

### Histological analysis of femur tissue

Femur tissue was collected immediately after the mice were euthanized and fixed in 4% paraformaldehyde. After incubation with a decalcification solution for 7 days, the tissue was embedded in paraffin, sectioned (5 μm thickness), and stained with hematoxylin and eosin and Giemsa. Sections were examined under a light-microscope equipped with a digital camera (Nikon, Japan).

### Micro-computed tomography

Femurs and tibias were collected from mice immediately after euthanasia. The structure of the trabecular and cortical regions of the femur, and the cortical regions of the tibia were evaluated by micro-computed tomography (micro-CT) using a micro-CT μCT50 (Scanco, Switzerland). Standard 3D microstructural analysis was performed to analyze parameters including the trabecular bone mineral density (BMD), bone volume fraction (BV/TV), trabecular thickness (Tb.Th), trabecular spacing (Tb.Sp), and trabecular number (Tb.N).

### Western blotting

MG63 cells were seeded into 6-well plates at a density of 2 × 10^5^ cells / well and then treated with 1, 2.5, or 5 μM AU for 3 h followed by 4 μM Dex or 200 μM H_2_O_2_ for 24 h. The cells were harvested and homogenized in RIPA lysis buffer (Sigma-Aldrich) containing 1% protease inhibitor cocktail and 2% phenylmethanesulfonyl fluoride (Sigma-Aldrich). Bone tissue lysates from AU-treated mice were generated by grinding tibias and fibulas from the mice into a powder under liquid nitrogen and homogenizing the powder in RIPA buffer as described. All lysates were then centrifuged at 10,000 rpm for 10 min. Protein concentrations were estimated using the BCA protein kit (Merck Millipore, USA). A total of 30 μg of protein was separated by 10%–12% SDS-PAGE and electrophoretically transferred onto nitrocellulose membranes (0.45 μm) (Bio Basic, Inc., Canada). The membranes were incubated with the following primary antibodies (1:2000 dilution) overnight at 4°C: collagen I (ab34710), OPN (ab91655), OCN (ab93876), osterix (ab22552), BMP2 (ab6285), integrin β1 (ab3167), Akt (ab131443), GAPDH (ab181602) (Abcam, USA), P-Akt (CST 4060S), and Smad1/5 (bs-4253R) (Bioss Inc., China). The following day, the membranes were washed and then incubated with horseradish peroxidase-conjugated secondary antibodies (1:2000) (Santa Cruz Biotechnology, USA). Protein bands was visualized using a BioSpectrum 600 imaging system (Bioss Inc., China). The pixel density was quantified using the Image J software (National Institutes of Health, USA).

### Reverse transcription-polymerase chain reaction (RT-PCR)

The RNA was isolated from MG63 cells using Trizol (Invitrogen, USA), and then synthesized by QuantScript RT Kit (Tiangen Biotech Co. Ltd., Beijing China). β-actin primers were used as an internal control. The conditions of PCR amplification were shown as follows: denaturation at 95 °C for 5 min, followed by 36 cycles at 95 °C for 45 s, 57 °C for 45 s and 72 °C for 45 s. The primer sequences are listed in Supplementary Table 1.

### Statistical analysis

Data are expressed as the mean ± standard deviation. One-way analysis of variance (ANOVA) followed by post-hoc Dunn’s multiple comparison tests was performed using SPSS 16.0 software (IBM Corporation, USA). *P* < 0.05 was considered significant.

## Supplementary Material

Supplementary Figures

Supplementary Table 1

Supplementary Results, Materials and Methods
