# Correction for: Aucubin exerts anti-osteoporotic effects by promoting osteoblast differentiation

**DOI:** 10.18632/aging.203365

**Published:** 2021-07-15

**Authors:** Yutong Li, Yongfeng Zhang, Xinrui Zhang, Wenqian Lu, Xin Liu, Min Hu, Di Wang

**Affiliations:** 1 Department of Orthodontics, School and Hospital of Stomatology, Jilin University, Changchun 130021, China; 2 School of Life Sciences, Jilin University, Changchun 130012, China; 3 Jilin Provincial Key Laboratory of Tooth Development and Bone Remodeling, Changchun 130021, China

**Keywords:** correction

Original article: Aging. 2020; 12:2226–2245.  . https://doi.org/10.18632/aging.102742

**This article has been corrected:** The authors replaced the HO-2 panel of the Western blot in **Figure 4B**, which was accidently mislabeled and duplicated with the HO-2 panel from Figure 2B. **Figure 5** was replaced because the original **Figure 5** was misprinted with an additional panel at the bottom, which was a partial copy of the above AU 45 mg/kg panel. In addition, the authors corrected **Supplementary Figure 3**, where the Nrf2 band was misplaced during figure preparation. All replacements were done using representative images from the original sets of experiments. These alterations do not affect the results or conclusions of this work.

The new **Figure 4, Figure 5** and **Supplementary Figure 3** are presented below.

**Figure 4:**
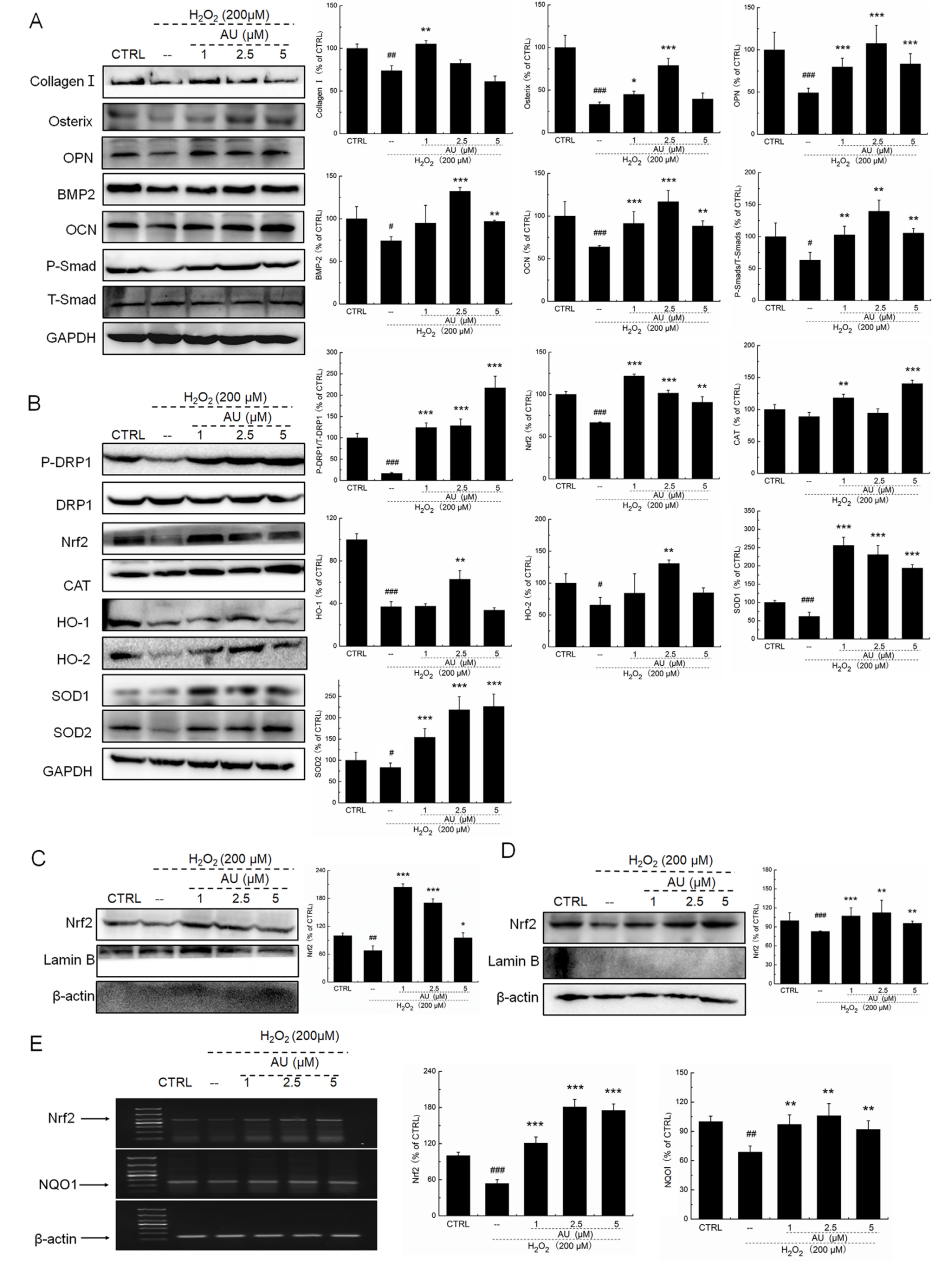
**AU protected the H_2_O_2_-caused MG63 cells apoptosis via regulation the Nrf2/HO-1 signaling.** (**A**) AU up-regulated the expression levels of osteoblast differentiation related proteins including Collagen I, Osterix, OPN, BMP2, OCN and P-Smad in MG63 cells exposed to H_2_O_2_. (**B**) AU increased the expression levels of proteins within the Nrf2/HO-1 signaling including P-DPR1, Nrf2, CAT, HO-1, HO-2, SOD-1 and SOD-2 in MG63 cells exposed to H_2_O_2_. AU enhanced the expression levels of Nrf2 in both (**C**) nucleus and (**D**) cytoplasm of MG63 cells exposed to H_2_O_2_. The quantification data of proteins were normalized by corresponding GAPDH, Lamin B, β-actin or total proteins, respectively (n=4). (**E**) AU increased the mRNA levels of Nrf2 and NQO-1 in MG63 cells exposed to H_2_O_2_. Marker size from top to bottom: 1000 bp, 700 bp, 500 bp, 400 bp, 300 bp, 200 bp and 100 bp. The data on quantified mRNA expression were normalized to the levels of β-actin (n=4). Data are expressed as mean ± S.D. and analyzed using a one-way ANOVA. # *P*<0.05, ## *P*<0.01 and ### *P*<0.001 *vs.* control cells, *P<0.05, **P<0.01 and ***P<0.001 *vs.* H_2_O_2_-exposed cells.

**Figure 5:**
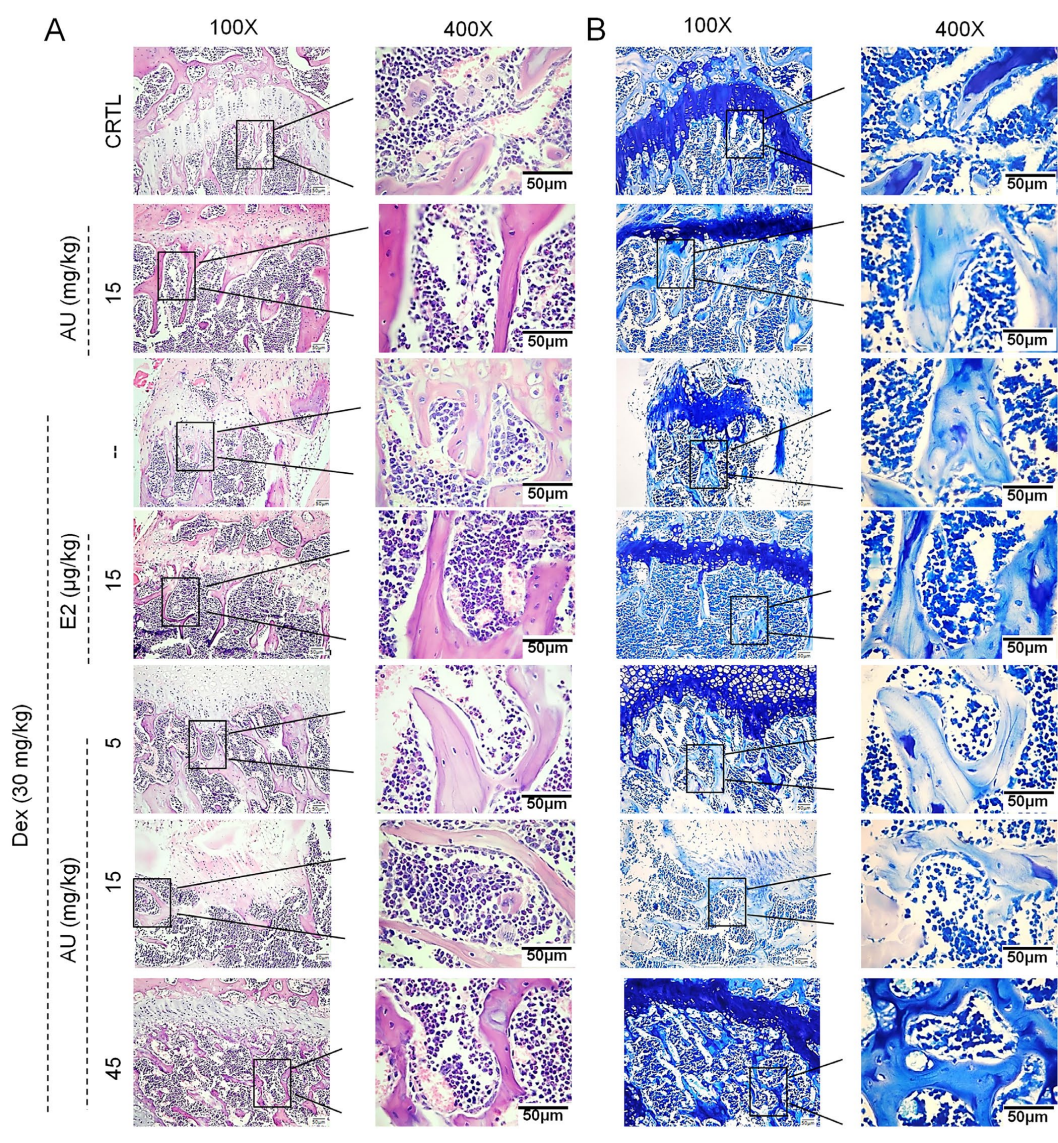
The effects of AU on the femoral histological changes of osteoporotic mice were detected by (**A**) H&E staining and (**B**) Giemsa staining (n=6).

**Supplementary Figure 3:**
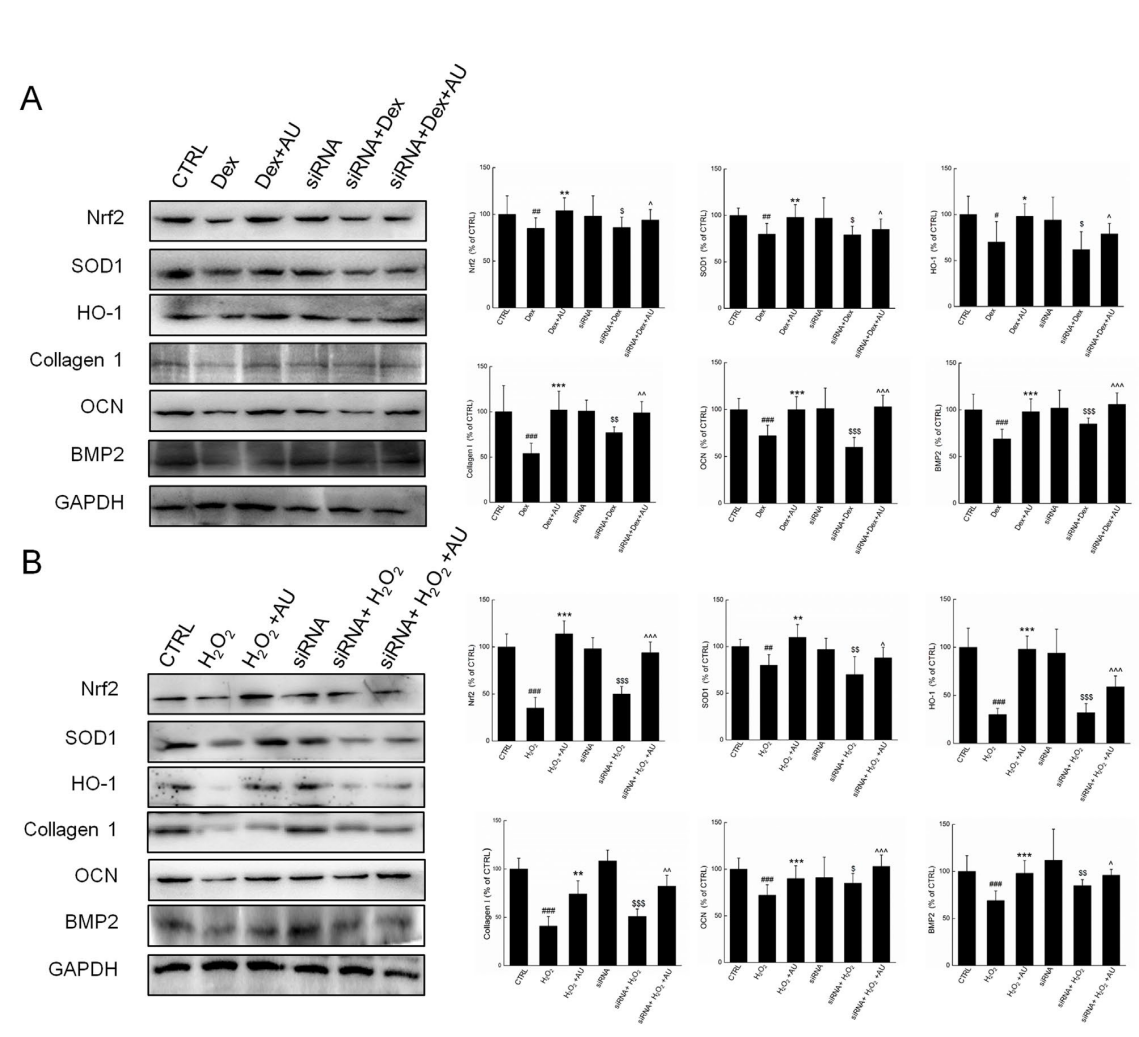
Negative siRNA transfection failed to influence the effects of AU on the protein expressions in (**A**) Dex and (**B**) H_2_O_2_ damaged MG63 cells. The quantification data of proteins were normalized by corresponding GAPDH, respectively, expressed as mean±S.D. (n=4) and analyzed using a one-way ANOVA. # P<0.05, ## P<0.01 and ### P<0.001 vs. control cells, *P<0.05, **P<0.01 and ***P<0.001 vs. Dex or H_2_O_2_-exposed cells, $ P<0.05, $$ P<0.01 and $$$ P<0.001 vs. negative siRNA transfected control cells, ^ P<0.05, ^^ P<0.01 and ^^^ P<0.001 vs. Dex or H_2_O_2_-exposed negative siRNA transfected cells.

