# Supplementary material for: Aucubin exerts anti-osteoporotic effects by promoting osteoblast differentiation
Source: Aging (Albany NY). 2020 Feb 5;12(3):2226–45. doi: 10.18632/aging.102742 (PMC7041723; doi:10.18632/aging.102742)
Supplement: Supplementary Figures [file aging-12-102742-s003..pdf]

SUPPLEMENTARY FIGURES

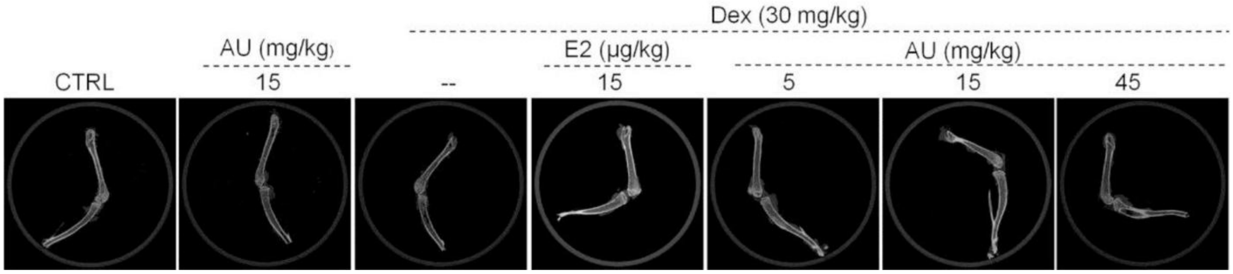

Supplementary Figure 1. AU increased the thickness of the cortical cortex and increased the consecutiveness of the cortial cortex.

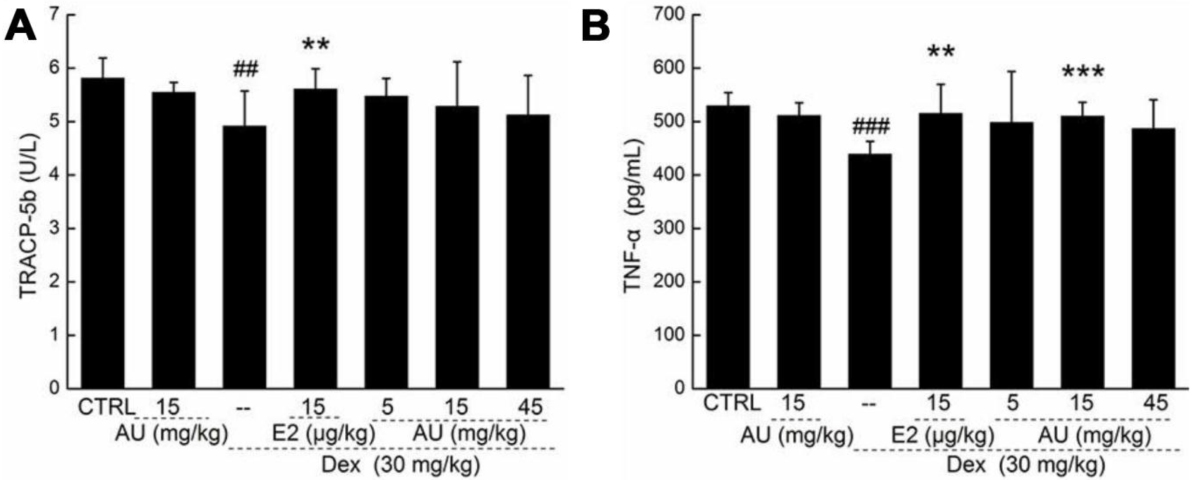

Supplementary Figure 2. The regulation effects of AU on the levels of (**A**) TRACP-5b and (**B**) TNF-α in serum of mice with osteoporosis. Data were expressed as mean ± S.E.D. (n=6) and analyzed using a one-way ANOVA. ## *P*<0.01 and ### *P*<0.001 *vs.* CTRL mice, ***P*<0.01 and ****P*<0.001 Dex-treated mice.

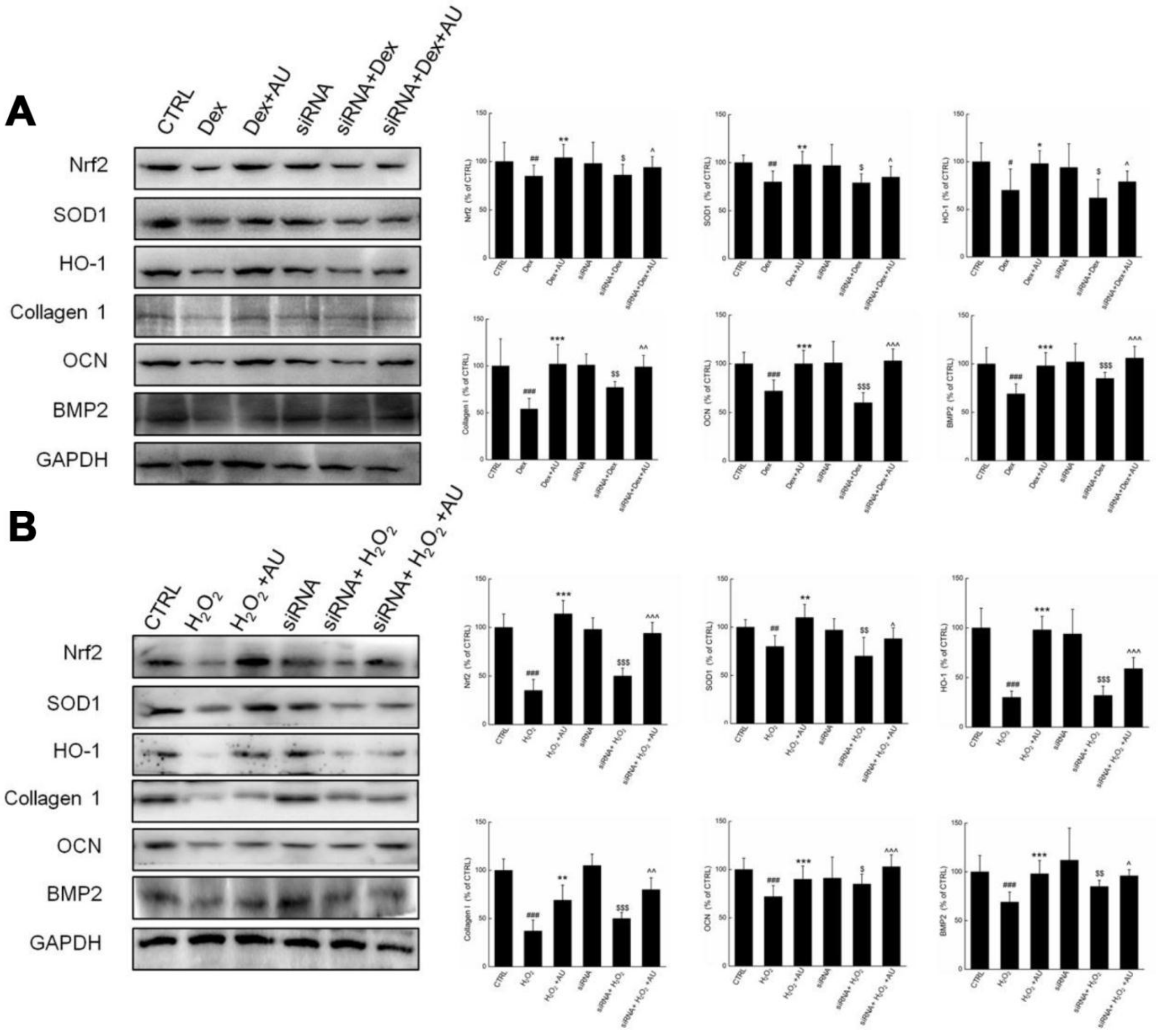

Supplementary Figure 3. Negative siRNA transfection failed to influence the effects of AU on the protein expressions in (**A**) Dex and (**B**) H_2O_2 damaged MG63 cells. The quantification data of proteins were normalized by corresponding GAPDH, respectively, expressed as mean ± S.D. (n=4) and analyzed using a one-way ANOVA. # *P*<0.05, ## *P*<0.01 and ### *P*<0.001 *vs.* control cells, **P*<0.05, ***P*<0.01 and ****P*<0.001 *vs.* Dex or H_2O_2-exposed cells, $ *P*<0.05, $$ *P*<0.01 and $$$ *P*<0.001 *vs.* negative siRNA transfected control cells, ^ *P*<0.05 and ^^^ *P*<0.001 *vs.* Dex or H_2O_2-exposed negative siRNA transfected cells.
