# Supplementary material for: Aucubin exerts anti-osteoporotic effects by promoting osteoblast differentiation
Source: Aging (Albany NY). 2020 Feb 5;12(3):2226–45. doi: 10.18632/aging.102742 (PMC7041723; doi:10.18632/aging.102742)
Supplement: Supplementary Table 1 [file aging-12-102742-s002..pdf]

SUPPLEMENTARY TABLE

Supplementary Table 1. The primer sequences used in RT-PCR.

Fragment size (bp)	Name	Sequence (5' to 3')
458	Nrf2-F	GTTGCCCACATTCCCAAACAA
	Nrf2-R	CTGGCATCATCAGTGGAGAGG
254	NQO1-F	GAGGTACTCGAATCTGACCTCTA
	NQO1-R	ACTCTCTCAAACCAGCCTTTC
137	β-actin-F	ATCGTGCGAGACATCAATG
	β-actin -R	TCGTTGCCTATTGTGATGAC
