# Supplementary material for: Aucubin exerts anti-osteoporotic effects by promoting osteoblast differentiation
Source: Aging (Albany NY). 2020 Feb 5;12(3):2226–45. doi: 10.18632/aging.102742 (PMC7041723; doi:10.18632/aging.102742)
Supplement: Supplementary Results, Materials and Methods [file aging-12-102742-s001..pdf]

Supplementary Results

AU enhanced the bone cortex thickness

Micro-CT was used to determine structural parameters of femur trabecular and cortical regions, as well as tibia cortical region. From the CT images, it is obvious that the cortical bone of the model group is thinner and the brightness is lower than that of the blank group, and the bone cancellous is sparse. The bone cortex was the thickest and the highest brightness in the positive control group. Compared with the model group, the cortical bone mass of the low-dose, middle-dose and high-dose groups significantly increased and the brightness increased, the network structure in the bone cancellous became dense, and the middle-dose group had the best affections (Supplementary Figure 1).

AU enhanced the expression of TNF-α and TRACP-5b

Different from E2, AU showed no significant effects on the levels of TRACP-5b (Supplementary Figure 2A). AU at 15 mg/kg and E2 at 15 μg/kg strongly enhanced the levels of TNF-α compared with model mice (*P*<0.01; Supplementary Figure 2B).

Negative siRNA failed to influence the effects of AU on protein expressions

In both Dex and H_2O_2 exposed MG63 cells, the negative siRNA transfection failed to influence the modulatory effects of AU on the expressions of Nrf2, SOD1, HO-1, Collagen 1, OCN and BMP2 (Supplementary Figure 3A and 3B).

Supplementary Materials and Methods

Bone micro-computed tomography (micro-CT)

Mouse femur and tibia were taken immediately after mouse euthanasia. Micro-CT was used to determine structural parameters of femur trabecular and cortical regions, as well as tibia cortical region. Micro-CT was scanned bones to get CT images of the femur and tibia of mice.

Plasma cytokine detection.

Peripheral blood from the orbital venous plexus of the mice was collected into clean tubes after the last administration. Blood samples were centrifuged for 10 minutes at 3,000 rpm after 30 minutes stationary at room temperature. Suck up the plasma which is supernatant into another clean tubes. Enzyme-linked immunosorbent assay (ELISA) kits were applied to determine the levels of plasma cytokines including TNF-α (YY02868B) and TRACP-5b (YY03144B). Both of the procedures were performed according to the kit manufacturer's protocol.

Transfection of negative siRNA

MG63 cells were plated in 6-well plates (2×10^5 cells/well) and were transiently transfected using the negative siRNA (20 nM, R10035.4, RIBBIO, Guangzhou, China) and the RNAiMAX reagent in riboFECT^{TM}CP Reagent, according to the manufacturer's specification (RIBBIO, Guangzhou, China). The transfected cells were exposed to 2.5 μM of AU for 2 h. Subsequently, 4 μM of Dex or 200 μM of H_2O_2 was added and the cells were incubated for a further 24 h. The collected cells were used to detect the protein expressions including Nrf2, SOD1, HO-1, Collagen 1, OCN, BMP2 and GAPDH.
